# Prevention of Neural Tube Defects by Folic Acid Supplementation: A National Population-Based Study

**DOI:** 10.3390/nu12103170

**Published:** 2020-10-16

**Authors:** Benoît de la Fournière, Ferdinand Dhombres, Paul Maurice, Sabine de Foucaud, Pauline Lallemant, Michel Zérah, Lucie Guilbaud, Jean-Marie Jouannic

**Affiliations:** 1 Fetal Medicine Department, Sorbonne University, AP-HP, Armand Trousseau Hospital, National Rare Disease Centre for Spinal and Vertebral Anomalies (MAVEM CENTER), 75012 Paris, France; delaf@hotmail.fr (B.d.l.F.); ferdinand.dhombres@aphp.fr (F.D.); paul.maurice@aphp.fr (P.M.); sabine.defoucaud@mavem.fr (S.d.F.); lucie.guilbaud@aphp.fr (L.G.); 2 INSERM U1142 (LIMICS), Sorbonne University, 75005 Paris, France; 3 Pediatric Rehabilitation Department, Sorbonne University, AP-HP, Armand Trousseau Hospital, National Rare Disease Centre for Spinal and Vertebral Anomalies (MAVEM CENTER), 75012 Paris, France; pauline.lallemant@aphp.fr; 4 Pediatric Neurosurgery Department, Paris University, APHP, Necker Hospital, National Rare Disease Centre for Spinal and Vertebral Anomalies (MAVEM CENTER), 75015 Paris, France; michel.zerah@aphp.fr

**Keywords:** folic acid, folate, neural tube defect, spina bifida, myelomeningocele, birth defects, prevention

## Abstract

Folic acid supplementation is recommended for neural tube defect prevention during pregnancy. We conducted an observational, retrospective national registry study to determine the rate of dispensing of periconceptional folic acid after prescription in a sample of French women representative of the general population. Our study population (*n* = 186,061) was a representative sample of the French population, recorded in the Health Data System database on pharmacy dispensing of medication and mandatory reporting of pregnancy. Between 2006 and 2016, 14.3% of pregnant women had a prescription for folic acid supplementation during the month preceding conception and for the first 12 weeks of pregnancy. Of these prescriptions, 30.9% were issued before the start of pregnancy. This percentage was lower for first pregnancies. The rate of pharmacy dispensing during the preconception period increased progressively from 3.8% to 8.3% between 2006 and 2016. In France, the rate of pharmacy dispensing of periconceptional folic acid after medical prescription is very low and does not follow international recommendations. It seems essential to implement awareness-raising policies targeting the general population and physicians regarding effective periconceptional supplementation, particularly starting in the preconception period. Clarification of international recommendations and fortification of flour could improve the efficacy of folate supplementation at population level.

## 1. Introduction

Neural tube defects (NTDs) are severe congenital malformations, the prevalence of which is 1 in 1000 pregnancies in Europe—stable between 2006 and 2016 [1]. They include malformations of the brain, spine, and spinal cord: anencephaly, encephalocele, and open and closed spinal dysraphism [2]. There is no curative treatment for these conditions, some of which are lethal at birth, others can cause bladder and sphincter dysfunction, neurological impairment, orthopedic deformities, sensorimotor dysfunction, and cognitive impairment [2], which require long-term multidisciplinary care [3]. For the severe forms, the rate of termination of pregnancy ranged from 70.8% to 83.5% among European countries in 2016 [1].

Although the pathophysiology of NTDs is poorly understood, some risk factors have been identified, such as folic acid deficiency, treatment of the mother with some antiepileptics, maternal obesity, uncontrolled maternal diabetes prior to pregnancy, vitamin B12 deficiency [4,5], and certain genetic anomalies [2]. Several multicenter, randomized, controlled trials since 1991 have shown that the prevalence of NTDs can be reduced by periconceptional folic acid supplementation [6,7]. In the first such trial conducted in 33 centers (17 in the UK and 16 in six other countries) [6], folic acid supplementation initiated four weeks before conception reduced the prevalence of NTDs by 72% [6]. These findings subsequently led to the drawing up of international guidelines recommending folic acid supplementation for “All women, from the moment they begin trying to conceive until 12 weeks of gestation” [8], but without explicitly mentioning the importance of starting supplementation at least four weeks before conception in order to achieve the efficacy proven by the first studies. Given the problems of pregnancy planning, an alternative solution that affords better population coverage is mandatory folic acid fortification of flour for the whole population. This policy has substantially reduced the prevalence of NTDs, notably in North America [9].

The addition of vitamins to foods is authorized in the European Union (EU) [10], yet, despite this regulation, no EU country has introduced mandatory folic acid fortification of flour [11]. Most European countries, like France, recommend daily supplementation with 0.4 mg of folic acid, from the beginning of attempts to conceive, i.e., at least four weeks before conception, and during the first 12 weeks of pregnancy [8,12]. Since the implementation of these recommendations, there has been no decrease in the prevalence of NTDs in Europe [1]. This seems to be due to the failure to take supplementation, because the prescription is not filled or because of poor treatment adherence, and partly to lack of pregnancy planning [13,14]. However, large-scale studies of adherence to such supplementation are scarce and are based on data gathered from self-report questionnaires administered after childbirth [15,16]. In European countries, no study has focused on data on the prescription and pharmacy dispensing of folic acid on a national scale.

The main objective of our study was to determine the rate of pharmacy dispensing of folic acid during the periconceptional period, as per French and the WHO recommendations, in a population of French women representative of the general population. The secondary objectives were to determine the rate of pharmacy dispensing in the preconception period by year and by parity.

## 2. Materials and Methods

### 2.1. Design of the Study

This was an observational, retrospective cohort study of data for the 11-year period from 2006 to 2016 from the National Health Data System (SNDS), which records all medical services reimbursed by public health insurance in France.

### 2.2. Source of Data and Population

We used SNDS data on people covered by public health insurance for family physician services (*Echantillon Généraliste des Bénéficiaires* or EGB). This national database is established from two sources: the SNIIRAM (public health insurance information system) and the PMSI (program of medicalization of information systems). The SNIIRAM concerns all French citizens, all of whom are covered for treatment under the social security system. It includes all applications for reimbursement of outpatient care. The PMSI records all hospital discharge summary reports in France [17]. The EGB comprises anonymous information on public health insurance beneficiaries and on the health services they have received as outpatients and during hospitalization, such as data on the dispensing of reimbursed medication following a medical prescription [17]. The EGB is a permanent sample representative of 1/97 of the French population covered by public health insurance, with random constitution of the sample depending on individual social security numbers by 5-year age bracket and sex [17,18].

### 2.3. Data Collected

Patients who had a pregnancy during the study period were identified in the EGB sample by their pregnancy, reporting of which is obligatory in France before 16 weeks and which includes the pregnancy start date, allowing to calculate gestational age and to distinguish pre-conception from post-conception supplementation. In the event of multiple reports of a pregnancy, we used the first reported pregnancy start date. Any new report of pregnancy for a given patient had to be separated by an interval of at least 3 months. One or more pregnancies could be recorded for a given patient during the study period. In order to describe our population, we also collected the following available patient data: age at pregnancy start date, previous pregnancy count and type of health coverage (public universal health coverage, mutual health coverage, private health insurance coverage and coverage by other institutions).

For data on the dispensing of folic acid after its prescription, we searched all dosage forms at doses of 0.4 or 5 mg reimbursed by public health insurance after medical prescription and available on the market during the study period, using their respective unique identifiers (CIP codes). Dispensing of the medication could correspond to a first prescription or to a renewal of a prescription. Dispensing could not exceed one box of up to 30 tablets per month.

Taking into account French and the WHO recommendations regarding folic acid supplementation, we defined a periconceptional period of dispensing as between 8 weeks before the pregnancy start date up to week 12 of pregnancy, so as to avoid overlooking anticipated prescriptions and to cover adherence during the month preceding pregnancy. The preconceptional period of dispensing corresponded to an 8-week period before the pregnancy start date up to the day before the start date.

### 2.4. Statistical Analysis

The main outcome measure was the rate of pharmacy dispensing of folic acid at doses of 0.4 and 5 mg during the periconceptional period, following medical prescription, for each pregnancy. The secondary outcome measure was the rate of preconceptional dispensing of folic acid for all women in our sample, according to parity and year.

The mean rates of dispensing in the peri- and preconceptional periods were calculated using SAS^®^ Enterprise Guide^®^ version 7.15 HF8 (7.100.5.6214) (SAS Institute Inc., Cary, NC, USA) and Microsoft^®^ Excel^®^ (Version 16.36)(Microsoft Corporation, Redmond, WA, USA), with a 95% confidence interval. The analysis was done for the whole study period (2006–2016), for each year, and depending on parity.

### 2.5. Details of Ethics Approval

We did not request ethical approval for our analysis of anonymized and aggregate data from the EGB sample, because the study did not involve human subjects. These data were from the French public health insurance database, secure and personal access to which is open for the French National Institute of Health and Medical Research (INSERM) by a direct connexion to the National Health Data System (SNDS), and was authorized by the French Data Protection Authority (CNIL agreements AT/CPZ/SVT/JB/DP/CR05222O of 14 June 2005 and DP/CR071761 of 28 August 2007).

## 3. Results

**Figure 1:**
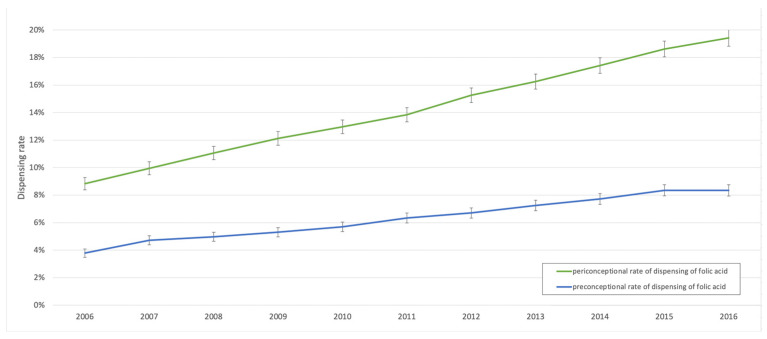
Annual rate of dispensing of folic acid in the preconceptional and periconceptional periods from 2006 to 2016, in a sample representative of the French population including 186,061 pregnancies.

From 2006 to 2016, 186,061 pregnancies were reported in the EGB (annual mean = 16,915 [16,340–17,489; 95% CI]). The characteristics of the population are detailed in Appendix A. In 26,601 of these pregnancies, folic acid was dispensed periconceptionally, so the mean rate of periconceptional dispensing was 14.3% (14.1–14.5; 95% CI). This rate progressively increased from 8.8% to 19.4% between 2006 and 2016 (Figure 1). Five different dosage forms were dispensed: SPECIAFOLDINE^®^ 0.4 and 5 mg, ACIDE FOLIQUE CCD^®^ 0.4 and 5 mg, and ACIDE FOLIQUE ARROW^®^ 5 mg.

**Figure 2:**
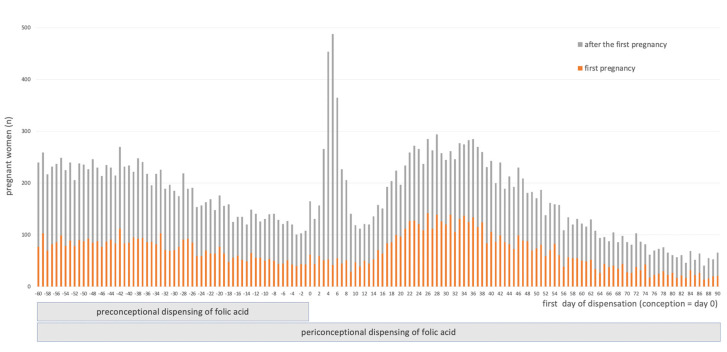
First day of dispensing of folic acid during the periconceptional period among 26,601 pregnancies in which a prescription enabled respect of French and international recommendations.

The date of the first dispensing of folic acid corresponded on average to day 5.7 of pregnancy (5.4–5.9; 95% CI) and was before the start of pregnancy in 30.9% of cases. The day of the first dispensing during the periconceptional period for these 26,601 pregnancies is shown in Figure 2.

Folic acid was dispensed preconceptionally to 11,819 (annual mean = 1074 [874–1275; 95% CI]) of the patients in our population, so the rate of preconceptional dispensing was 6.4% (6.2–6.5%; 95% CI). The rate of preconceptional dispensing of folic acid was 5.6% for first pregnancies (5.5–5.7%; 95% CI) and 8.7% for subsequent pregnancies (8.6–8.8%; 95% CI). Dispensing was primarily of 0.4 mg dosage forms (Table 1). From 2006 to 2016, the rate of preconceptional dispensing of folic acid increased from 3.8% to 8.3% of pregnancies (Table 2). Figure 1 shows the rates of periconceptional and preconceptional dispensing of folic acid.

## 4. Discussion

### 4.1. Main Findings

Using data from a sample representative of the French population from 2006 to 2016, we observed that only 14.3% of pregnant patients received a prescription followed by dispensing of folic acid within a timeframe enabling adherence to French and international recommendations. We also noted an increase in dispensing over the period of our study, from 8.84% in 2006 to 19.43% in 2016. If we consider the proportion of women who filled a prescription for folic acid in the eight weeks before the start of pregnancy, thus enabling supplementation in line with the early studies showing that folic acid intake at least four weeks before conception reduced the incidence of NTDs, the coverage was even lower, with an average rate of dispensing of 6.4%. This rate was even lower (5.9%) among first pregnancies.

### 4.2. Strengths and Limitations

Ours is the first observational study in a European country to present objective data on the prescription and dispensing of folic acid in a sample representative of the general population. The power of our study is considerable since we included 186,061 pregnancies recorded over an 11-year period. We exhaustively collected data on the dispensing of folic acid in its dosage form reimbursed by public health insurance, whether for a first prescription or a renewal. Although the EGB represents only 1/97 of the French population covered by public health insurance, this sample is constituted randomly and such that the age and sex distributions are very close to those of the French population [18], which limits bias in selection of regional patient cohorts. There is, therefore, no reason to suppose that the rate of dispensing in the general population of pregnant women differs from that in our sample.

Moreover, these data are objective and behavioral, unlike those of the largest studies, which used self-report data from questionnaires completed in the postnatal period. These studies also involved much smaller patient samples, such as 14,156 patients in Camier et al., in 2019 [16] and 12,646 patients in Tort et al in 2013 [15]. Only the latter study [15] individualized preconceptional supplementation, whereas the former did not distinguish patients who had preconceptional supplementation from those who received only postconceptional supplementation [16].

The main limitation of our study is that we used data on pharmacy dispensing and not data on adherence to treatment. The rate of pharmacy dispensing corresponds neither to the rate of prescription by caregivers nor to the rate of treatment adherence. In effect, a patient can receive a prescription but not fill it at the pharmacy; or, she can fill the prescription but not take the medication. Nonetheless, dispensing data are the best proxy of the intake of prescribed medication. Data on treatment adherence are only available when the taking of medication is directly monitored by a caregiver, which is not feasible in the context of prevention on a population scale.

We may have underestimated the real folic acid supplementation as we did not count the taking of supplements bought over the counter. However, to our knowledge, there is no database that can be used to determine such over-the-counter use by pregnant women. Interestingly, at the population level in France, the total number of unit sales of the two main dosage forms (SPECIAFOLDINE^®^ 0.4 and ACIDE FOLIQUE CCD^®^ 0.4) was estimated between 28% and 36% over-the-counter in 2006, with a decrease down to 8% to 0% in 2016 (source: EGB and data from the global national commercial pharmaceutical industry database GERS^®^). Consequently, the remaining sales of these two preparations were following a prescription, and therefore being accounted for within the public health insurance national database. Thus, the coverage of the public health insurance national database comprised most folic acid dispensations, up to 92–100% in 2016. Accordingly, we can estimate that the over-the-counter use by pregnant women remains limited.

### 4.3. Interpretation

NTDs arise during the fourth week of pregnancy [2]. To be effective, folic acid supplementation must be started daily at appropriate doses at least four weeks before conception [12]. There is a very low rate of preconceptional dispensing in France in the eight weeks preceding pregnancy. One explanation could be that half of pregnancies are unplanned, as shown by Finer and Zolna in the USA [14]. This could be taken as an argument for mandatory folate fortification, notably of certain foods, as is already the case in some countries [11]. Furthermore, international recommendations should be clarified if we wish to increase preventive coverage, and the notion of starting supplementation from the beginning of attempts to conceive should be replaced by starting at least one month before conception.

Our findings point to poorer adherence to WHO recommendations [8] in France than in some other European countries. For instance, in the Netherlands, studies have shown better adherence to recommendations, with self-report data from questionnaires indicating adherence of 37% to 51.6% in the preconceptional period [19,20]. However, the prevalence of NTDs in the Netherlands increased from 0.88 to 1.4/1000 pregnancies between 2006 and 2016 [1]. In Ireland [21], self-report questionnaires indicate that periconceptional adherence decreased from 45.1% in 2009 to 43.1% in 2013 (*n* = 42,042), with a parallel sharp increase in the prevalence of NTDs from 0.59 to 1.31/1000 pregnancies from 2006 to 2016 [1]. These results raise the question of the validity of the rates of adherence self-reported by patients and/or of the efficacy of prevention by folic acid in these populations. Belgium is the only country to have recorded a large drop in the rate of NTDs since the WHO recommendations, from 1.31 to 0.71/1000 pregnancies between 2006 and 2016 [1]. In a Belgian study [22], 41.2% of the women included (*n* = 1311) reported having taken preconceptional supplementation and 61% had a first-trimester blood folate concentration above 400 ng/mL, in line with WHO recommendations [23]. These figures suggest that adherence to WHO recommendations can significantly reduce the prevalence of NTDs in the general population [22], even in the absence of folic acid fortification of foods, notably flour.

The recommended preventive measures by means of folic acid supplementation in women planning a pregnancy therefore seem generally insufficient or ineffective in most European countries. The prevalence of NTDs in Europe did not decrease between 2006 and 2016, with respective rates of 1.14/1000 and 1.32/1000 pregnancies [1]. Outside Europe, the failure of supplementation has led 59 countries to mandate folic acid fortification of flour [24]. This has led to a significant reduction in NTDs in all those countries [9]. For example, the incidence of NTDs has decreased in the USA and Canada from, respectively, 1.07 to 0.7 and 1.58 to 0.86 NTDs per 1000 live births [25,26]. Also in the USA and Canada, the population blood folate concentration rose after fortification [27].

The socioeconomic status has been reported as strongly associated with NTDs prevalence, thus questioning the actual contribution of food folate fortification in several countries [28,29,30]. However, mandatory fortification can be considered one of the most effective and cost-effective public health measures to date: the estimated annual economic gain in the USA is 603 million dollars [31]. In France, the estimated annual cost of adequate folic acid supplementation in the whole population is 14.5 million euros. This cost should be balanced against the cost of caring for the disabled, which over 70 years of life is estimated as 3.3 million euros per person.

Folic acid fortification is currently not in force in any EU countries [11]. This reluctance is perhaps linked to suspicion of side effects associated with folic acid supplementation or to the costs generated by these public health measures. Nonetheless, a putative association between folic acid and colorectal cancer has been rebutted [32], and the risks of masked vitamin B12 deficiency do not concern pregnant women at the doses used [32]. So, in view of these reassuring data, the Scientific Advisory Committee on Nutrition in the UK in 2019 supported the mandatory fortification of flour with folic acid, i.e., the legal requirement to add folic acid to flour, a measure which at the time of writing has not been implemented by the British government [33].

## 5. Conclusions

Our study demonstrates poor adherence in France to recommendations concerning periconceptional folic acid supplementation. Policy regarding this supplementation must therefore change. It appears necessary to increase awareness-raising campaigns among women aged 15 to 45 and among caregivers, so as to achieve coverage at least equivalent to that of Belgium. In addition, it is important that the recommendations emphasize more clearly the benefits of supplementation started at least one month before beginning of attempts to conceive. Another way to overcome the limits of a folic acid supplementation policy could be mandatory folic acid fortification of flour, given its proven beneficial impact in North America. If mandatory folic acid fortification of flour is introduced by the British government, changes in the prevalence of NTDs in the UK in the years ahead will be determinant in the balance of decisions across all European countries.

## Figures and Tables

**Table 1:** Rate of preconceptional dispensing of folic acid from 2006 to 2016, in 186,061 pregnancies.

	First Pregnancy *n* (%) [95% CI]	Second or Subsequent Pregnancy *n* (%) [95% CI]	All Patients *n* (%) [95% CI]
Rate of dispensing of folic acid 0.4 mg	4231 (6.61%) [6.41–6.80%]	4971 (4.07%) [3.96–4.19%]	9202 (4.95%) [4.85–5.04%]
Rate of dispensing of folic acid 5 mg	384 (0.60%) [0.54–0.66%]	2233 (1.83%) [1.75–1.91%]	2617 (1.41%) [1.35–1.46%]
Rate of dispensing of folic acid 0.4 mg and 5 mg	4615 (7.21%) [7.00–3.83%]	7204 (5.90%) [5.77–6.04%]	11,819 (6.35%) [6.24–6.46%]

**Table 2:** Number of pregnancies and rate of dispensing of folic acid in the preconceptional period, between 2006 and 2016 in France.

Year	Pregnancies	Pregnancies with Preconceptional Dispensing of Folic Acid	Rate of Preconceptional Dispensing of Folic Acid	
	*n*	*n*	%	95% CI
2006	15,139	574	3.79%	[3.49–4.10%]
2007	15,729	743	4.72%	[4.39–5.06%]
2008	16,417	816	4.97%	[4.64–5.30%]
2009	16,525	877	5.31%	[4.97–5.65%]
2010	17,328	989	5.71%	[5.36–6.05%]
2011	17,345	1101	6.35%	[5.98–6.71%]
2012	17,693	1187	6.71%	[6.34–7.08%]
2013	17,566	1274	7.25%	[6.87–7.64%]
2014	17,342	1339	7.72%	[7.32–8.12%]
2015	17,737	1481	8.35%	[7.94–8.76%]
2016	17,240	1438	8.34%	[7.93–8.75%]
**2006–2016**	**186,061**	**11,819**	**6.35%**	**[6.24**–**6.46%]**

## Appendix A

**Table A1:** Population Characteristics.

	2006	2007	2008	2009	2010	2011	2012	2013	2014	2015	2016	2006–2016
**Patients**												
N	13,361	13,761	14,352	14,355	14,920	14,774	14,875	14,682	14,540	14,931	14,509	**159,060**
**Age at conception**												
Median (year) ± SD	29 ± 5.42	29 ± 5.48	30 ± 5.50	30 ± 5.58	30 ± 5.60	30 ± 5.53	30 ± 5.50	30 ± 5.49	30 ± 5.46	30 ± 5.44	30 ± 5.44	**30 ± 5.50**
**Pregnancy counts**												
First pregnancy (*n*)	5.720	5.578	5.708	5.569	6.033	5.878	6.059	5.968	5.848	6.022	5.669	**64.052**
First pregnancy (%)	38%	35%	35%	34%	35%	34%	34%	34%	34%	34%	33%	**34%**
Second or subsequent pregnancy (*n*)	9419	10,151	10,709	10,956	11,295	11,467	11,634	11,598	11,494	11,715	11,571	**122,009**
Second or subsequent pregnancy (%)	62%	65%	65%	66%	65%	66%	66%	66%	66%	66%	67%	**66%**
All pregnancy (*n*)	15,139	15,729	16,417	16,525	17,328	17,345	17,693	17,566	17,342	17,737	17,240	**186,061**
**Health coverage**												
Public universal health coverage	8%	8%	8%	8%	8%	8%	8%	9%	9%	9%	9%	**8%**
Mutual health coverage	68%	66%	66%	65%	64%	64%	63%	62%	62%	63%	62%	**64%**
Private health insurance coverage	14%	15%	16%	16%	17%	17%	18%	18%	18%	18%	19%	**17%**
Coverage by other institutions	10%	11%	11%	11%	11%	11%	11%	11%	11%	10%	10%	**11%**
